# Supplementary material for: Revealing pathogenesis-associated metabolites in Histoplasma capsulatum through comprehensive metabolic profiling
Source: mSystems. 2025 Feb 27;10(3):e00186-25. doi: 10.1128/msystems.00186-25 (PMC11915866; doi:10.1128/msystems.00186-25)
Supplement: Supplemental Figures — Fig. S1 to S3. [file msystems.00186-25-s0001.pdf]

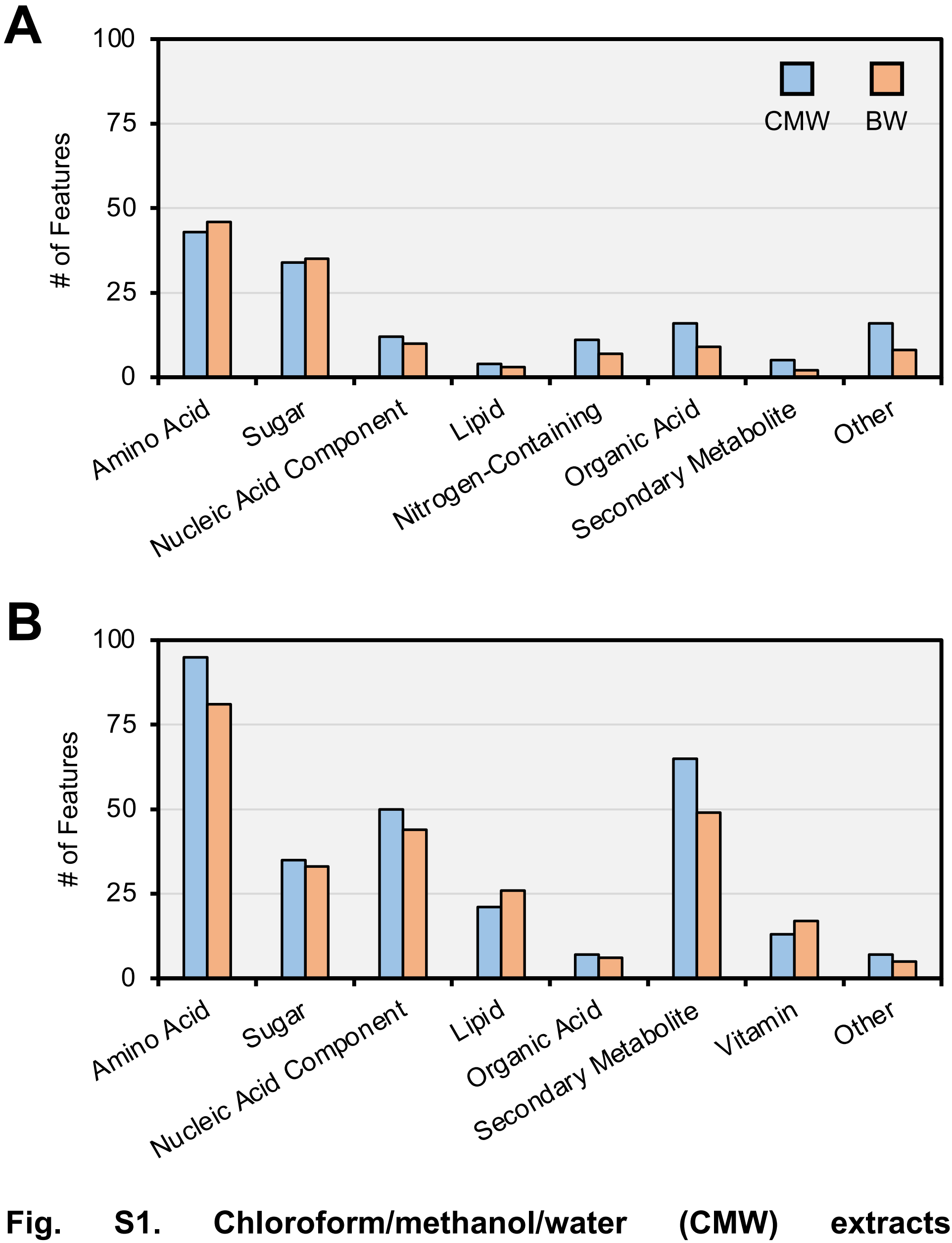

Fig. S1. Chloroform/methanol/water (CMW) extracts *Histoplasma* metabolome more efficiently than boiling water (BW). (**A**) Number of metabolites annotated per class of compound using GC-MS or (**B**) LC-MS/MS with a HILIC column in both ionization modes. Annotations have at least 70% spectral matching with GC-MS analysis or a total score of 1.1 or greater with LC-MS/MS analysis. Features that were found to be abundant within blanks, or with an average signal/noise less than 10, or duplicated annotations were excluded from this analysis.

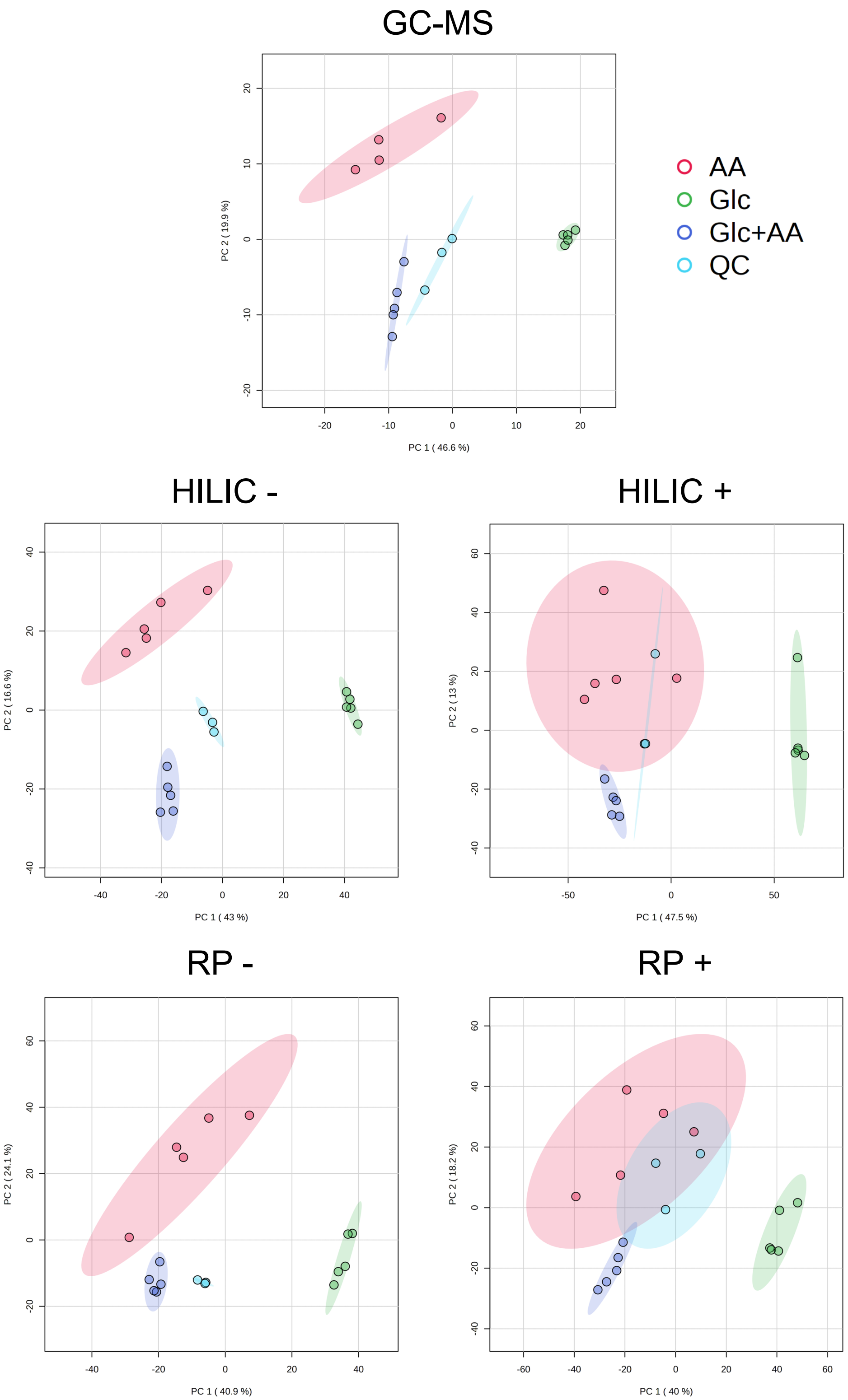

Fig. S2. *In vitro* growth of *Histoplasma* on glucose (Glc), amino acids (AA), or glucose + amino acids (Glc+AA) yields unique metabolomes for each condition. Quality controls (QC) represent mixture of all biological samples, and shaded regions represent 95% confidence intervals. Datasets displayed are from GC-MS, HILIC positive, HILIC negative, RP positive, and RP negative untargeted metabolomic analyses. Data reported are only blank filtered, meaning that features with a signal/noise ratio less than 10, unknown features, and duplicate annotations were kept for principal component analysis. n=3 for quality controls, n=4 for GC-MS amino acid supplemented samples, and n=5 for all other datasets and conditions.

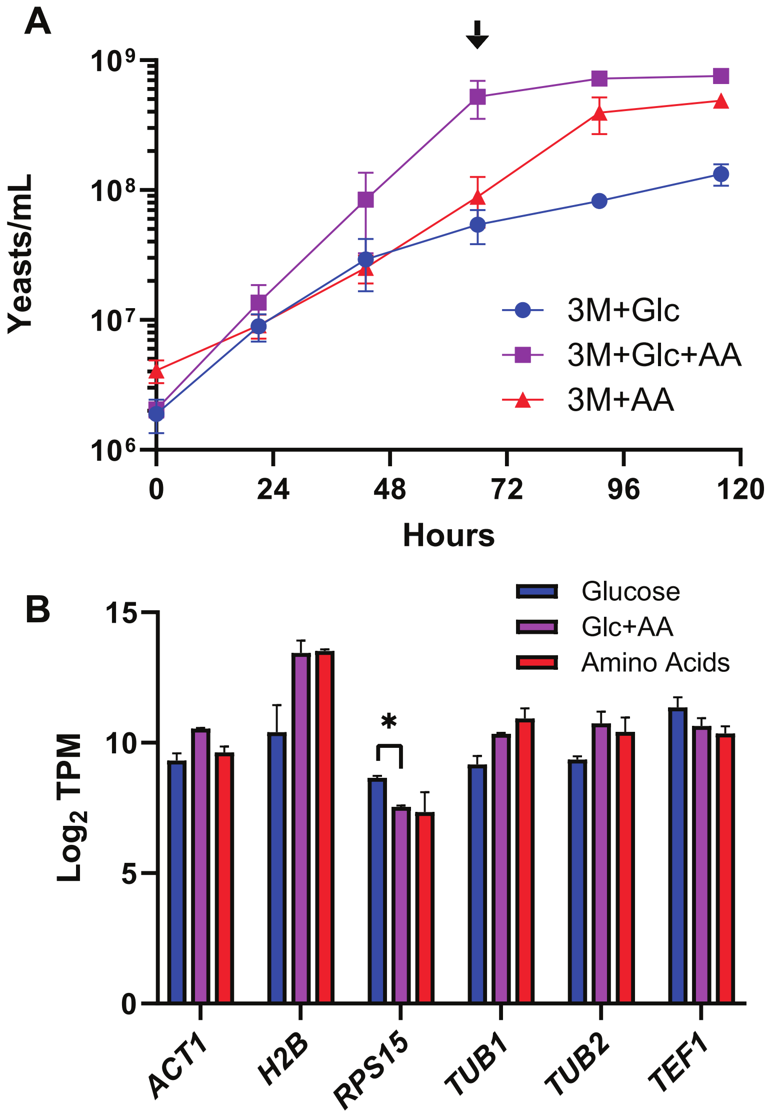

Fig. S3. Growth curves and expression of *Histoplasma* housekeeping genes in respective growth conditions. (**A**) Growth of yeasts in defined media containing glucose and ammonium sulfate as the carbon/nitrogen source (3M+Glc, blue circles), media containing a mixture of amino acids as the carbon/nitrogen source (3M+AA, red triangles), or a 1:1 mixture of both media (3M+Glc+AA, purple squares). Yeasts were collected for RNA extraction and metabolite isolation at 68 hours of incubation, denoted by the black arrow. (**B**) TPM (transcripts per million [reads]) for *Histoplasma* housekeeping genes in defined media containing glucose and ammonium sulfates as the carbon/nitrogen source (Glucose, blue bars), media with a mixture of amino acids as the carbon/nitrogen source (Amino Acids, red bars), or a 1:1 mixture of both media (Glc +AA, purple bars). Housekeeping genes analyzed included actin (ACT1), histone 2B (H2B), Rps15 small ribosomal protein subunit (RPS15), alpha-tubulin (TUB1), beta-tubulin (TUB2), and translational elongation factor EF-1 alpha (TEF1).
